# Liver-specific deletion of *Eva1a/Tmem166* aggravates acute liver injury by impairing autophagy

**DOI:** 10.1038/s41419-018-0800-x

**Published:** 2018-07-10

**Authors:** Xin Lin, Ming Cui, Dong Xu, Dubeiqi Hong, Yan Xia, Chentong Xu, Riyong Li, Xuan Zhang, Yaxin Lou, Qihua He, Ping Lv, Yingyu Chen

**Affiliations:** 1 Department of Immunology, Peking University School of Basic Medical Science; Key Laboratory of Medical Immunology, Ministry of Health, Peking University Health Sciences Center, 100191 Beijing, China; 2 Department of Cardiology, Peking University Third Hospital, 100191 Beijing, China; 3 Department of Clinical Laboratory, Peking University First Hospital, 100034 Beijing, China; 4 Medical and Healthy Analytical Center, Peking University, 100191 Beijing, China

## Abstract

Acute liver failure (ALF) is an inflammation-mediated hepatocellular injury process associated with cellular autophagy. However, the mechanism by which autophagy regulates ALF remains undefined. Herein, we demonstrated that Eva1a (eva-1 homolog A)/Tmem166 (transmembrane protein 166), an autophagy-related gene, can protect mice from ALF induced by d-galactosamine (D-GalN)/lipopolysaccharide (LPS) via autophagy. Our findings indicate that a hepatocyte-specific deletion of *Eva1a* aggravated hepatic injury in ALF mice, as evidenced by increased levels of alanine aminotransferase (ALT) and aspartate aminotransferase (AST), myeloperoxidase (MPO), and inflammatory cytokines (e.g., TNFα and IL-6), which was associated with disordered liver architecture exhibited by *Eva1a*^−/−^ mouse livers with ALF. Moreover, we found that the decreased autophagy in *Eva1a*^−/−^ mouse liver resulted in the substantial accumulation of swollen mitochondria in ALF, resulting in a lack of ATP generation, and consequently hepatocyte apoptosis or death. The administration of Adeno-Associated Virus Eva1a (AAV-Eva1a) or antophagy-inducer rapamycin increased autophagy and provided protection against liver injury in *Eva1a*^−/−^ mice with ALF, suggesting that defective autophagy is a significant mechanism of ALF in mice. Collectively, for the first time, we have demonstrated that Eva1a-mediated autophagy ameliorated liver injury in mice with ALF by attenuating inflammatory responses and apoptosis, indicating a potential therapeutic application for ALF.

## Introduction

Acute liver failure (ALF) is a clinical syndrome that involves hepatocellular apoptosis and necrosis^1^. Common causes of ALF include viral hepatitis, hepatic ischemia-reperfusion injury, drug overdose, idiosyncratic drug reactions, and the ingestion of toxic substances^2^. In addition, ALF is associated with a very high mortality rate and has a poor prognosis^3^. Thus, liver transplantation is the only therapy for end stage of ALF that has proven to be beneficial^4^. Although the nature of ALF has been widely studied, the mechanisms are not completely understood.

The co-administration of the hepatocyte-specific transcriptional inhibitor, d-galactosamine (d-GalN), and the endotoxin, lipopolysaccharide (LPS) is an established model for studying ALF in mice. Moreover, this model has been widely used to study the mechanisms of ALF pathogenesis and identify novel therapeutic drugs^5^. d-galactosamine (d-GalN) is a specific hepatotoxic agent which leads to a depletion of hepatic UTP, followed by the cessation of macromolecule biosynthesis, followed by alterations in the structure and function of the plasma membrane, eventually causing cellular damage and death. d-GalN treatment leads to a thousand fold increase in the susceptibility to the lethal effects of LPS^6^. Upon stimulation with LPS in this model, Kupffer cells and infiltrating macrophages in the liver secrete pro-inflammatory cytokines, including IL-1β, IL-6, and TNF-α. Among these cytokines, TNF-α is a particularly important mediator that induces hepatocyte apoptosis and liver failure. TNF-α is primarily responsible for neutrophil recruitment into the liver sinusoids and is also the main inducer of various adhesion molecules and chemokines in hepatocytes^7^.

Macroautophagy (referred to hereafter as autophagy) is a highly evolutionarily conserved cellular process in which cytoplasmic components are sequestered within double-membrane-bound compartments known as the autophagosome, which delivers them to the lysosome for degradation^8^. Evidence has shown that liver autophagy contributes to basic hepatic functionality, including glycogenolysis, gluconeogenesis, lipolysis, protein catabolism, and β-oxidation^9^. Quality and quantity control of mitochondria and peroxisomes through selective autophagy also directly regulates the features of hepatic metabolism^9^. Accumulated results show that autophagy also plays an important role in liver pathophysiology, including liver protein aggregate-related disease, steatohepatitis, hepatocyte cell death, hepatitis virus infection, and hepatocellular carcinoma^10^. Recent reports reveal that autophagy is involved in the development of ALF^1,11–13^; however, the precise relationship between autophagy and ALF remain undefined.

EVA1A (Eva-1 homolog A), also known as TMEM166 (transmembrane protein 166) or FAM176A (family with sequence similarity 176), is a lysosome and endoplasmic reticulum-associated protein that can regulate cellular autophagy and apoptosis^14^. Previous studies have demonstrated that EVA1A is expressed in a cell- and tissue- specific manner, and is significantly downregulated in many types of human tumors^15^. Moreover, EVA1A overexpression inhibits tumor proliferation by both autophagy and apoptosis^16–18^. Studies investigating the molecular mechanism of this inhibition proved that EVA1A interacts with the WD repeats of ATG16L1 through its C-terminal, promotes ATG12–ATG5/ATG16L1 complex recruitment to the autophagic membrane, and enhances the formation of the autophagosome^19^. Other research has demonstrated that EVA1A/TMEM166 is a key player in the induction of C/EBPα-mediated autophagy and protects against starvation in mouse hepatocellular carcinoma^20^. Additionally, EVA1A-mediated autophagy may also play an important role in the generation of newborn neurons^21^, cardiac remodeling^22^, and HBV replication^23^, indicating its functional diversity and complexity. However, the role of EVA1A in liver disease remains unknown.

In the present study, we generated *Eva1a* knockout mice to investigate the role of EVA1A in ALF. Our results show that the *Eva1a*^*−/−*^ mice exhibited more severe liver injury following ALF, which was accompanied by impaired autophagy. Pretreatment with rapamycin or AAV-Eva1a provided protection against liver injury in *Eva1a*^−/−^ mice. Thus, our study highlighted a novel role of Eva1a-mediated autophagy in association with liver pathophysiology.

## Results

### EVA1A expression profile in mice with ALF induced by D–GalN/LPS

**Fig. 1:**
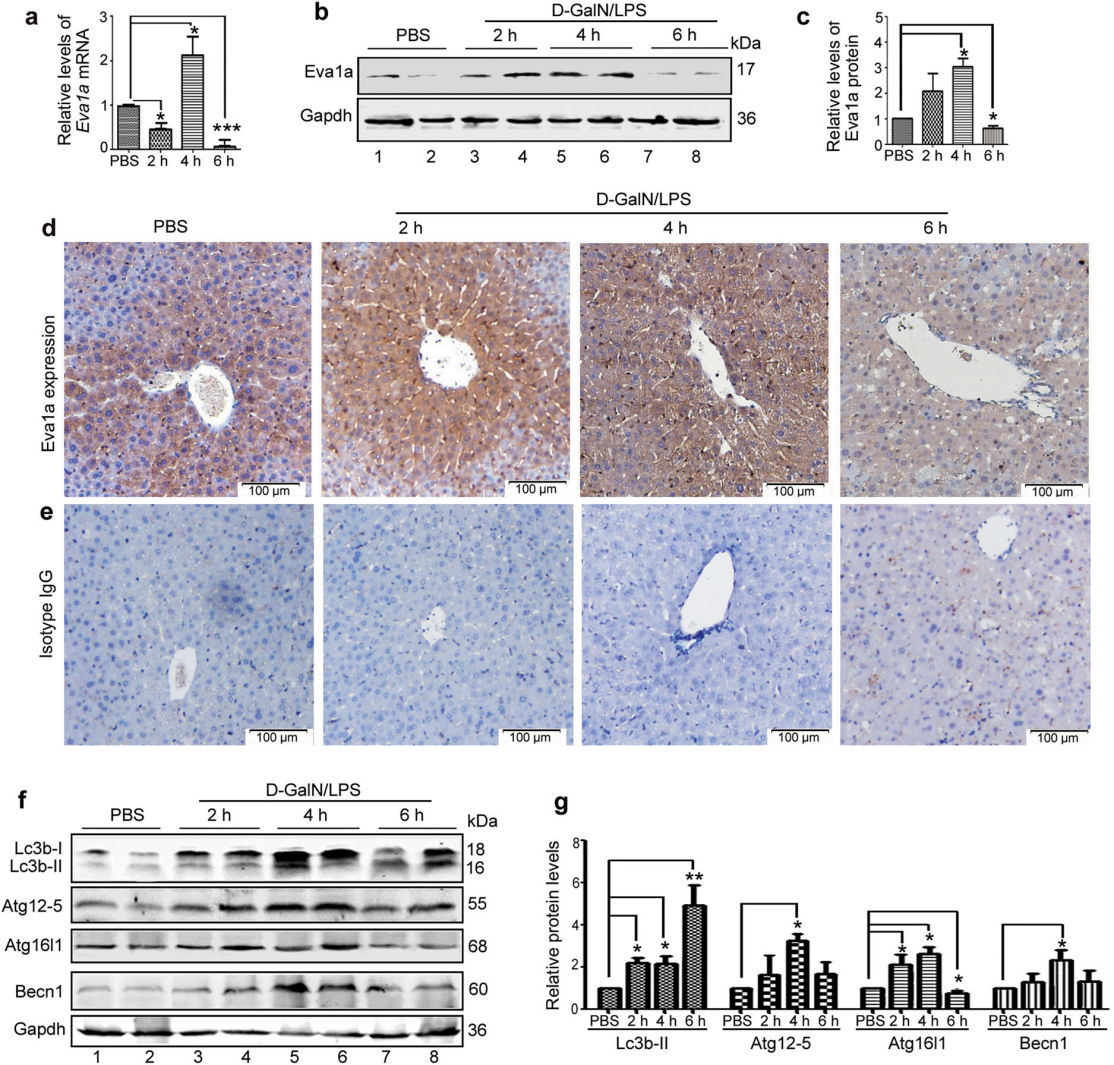
EVA1A expression profile and autophagy in mice with D–GalN/LPS-induced acute liver failure. Mice were intraperitoneally injected with d-GalN (350 mg/kg) and LPS (30 μg/kg) and killed at 2, 4, or 6 h (*n* = 5). The mice in the control group (*n* = 4) were injected with PBS only. **a** The relative levels of *Eva1a* mRNA were measured by qRT-PCR in the livers (*P < 0.05, ***P < 0.001, *n* = 4). **b** The levels of Eva1a protein were measured by Western blot in the livers. A representative blot from two samples of every group is shown. **c** The quantification of Eva1a levels relative to Gapdh treated as described in **b**. The average value of PBS-treated mice was normalized to 1 (*P < 0.05, *n* = 4). **d** Eva1a expression was detected by an immunohistochemical analysis of the liver tissues. Scale bar = 100 µm. **e** Isotype IgG staining of liver tissues. **f** The levels of Lc3b, Atg12-5, Atg16l1, Becn-1, and Eva1a were measured by a Western blot assay of the livers. A representative blot from two samples of every group is shown. **g** Quantification of the indicated protein levels relative to Gapdh treated as described in (**f**). The average value for the PBS-treated mice was normalized to 1 (*P < 0.05, **P < 0.01, *n* = 4)

We constructed a mouse model of ALF following d-GalN/LPS injection and dynamically evaluated the expression of Eva1a at 2, 4, or 6 h, compared to control mice. Data from quantitative real-time PCR suggested that the level of *Eva1a* mRNA decreased at 2 h, increased at 4 h, and then declined again at 6 h (Fig. 1a). The change in Eva1a protein expression was in agreement with that of the *Eva1a* mRNA. Data from western blot indicated that the levels of Eva1a protein was increased at 2–4 h, but declined at 6 h (Fig. 1b, c). Immunohistochemical experiments further proved this expression tendency of Eva1a (Fig. 1d). As a negative control, isotype IgG failed to stain hepatocytes (Fig. 1e). Similar to Eva1a expression, a tendency was observed for the key autophagy proteins, Atg12-5, Atg16l1, and Beclin1, to change during the same time period in mice with ALF (Fig. 1f, g). However, the accumulation of Lc3b-II gradually increased from 2 h to 6 h (Fig. 1f, g). These data suggest that Eva1a-mediated autophagy might be involved in d-GalN/LPS-induced ALF.

### Generation of liver-specific *Eva1a*^*−/−*^ mice

**Fig. 2:**
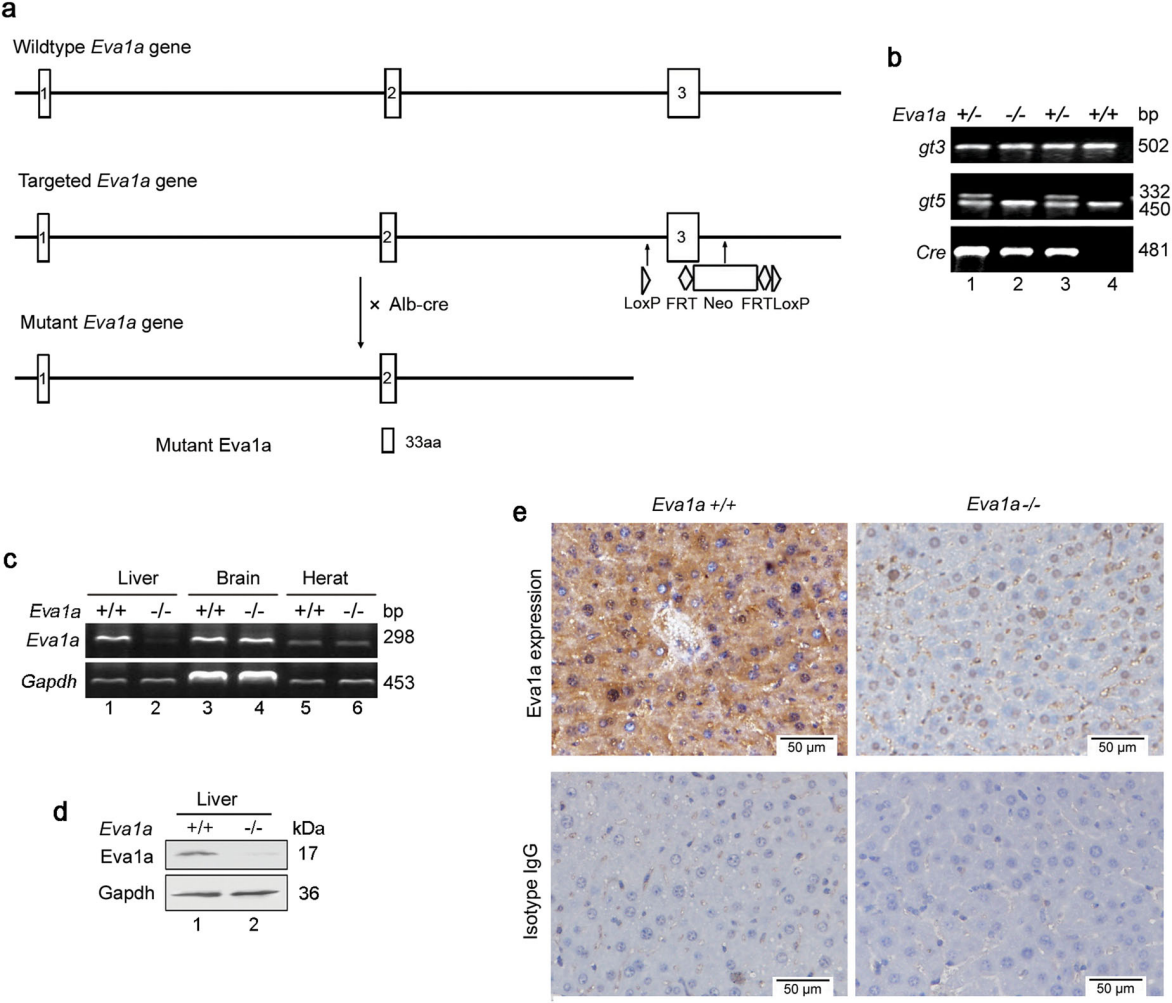
Generation of liver-specific *Eva1****a***^**−/−**^ mice. **a** Scheme used to generate *Eva1a* deficient mice. **b** Genomic DNA was extracted from mouse tails and analyzed by PCR. **c** Representative RT-PCR results showing endogenous *Eva1a* expression in different tissues from Eva1a^f/f^ and *Eva1a*-deficient mice. **d** The levels of Eva1a protein were detected by Western blot assays. **e** Eva1a expression was detected by immunohistochemical analysis. Isotype IgG staining was used as a negative control. Scale bar = 50 µm

Consistent with previous reports, the RT-PCR results showed that *Eva1a* mRNA was widely expressed in mouse tissues^22^, and was highly expressed in the adult liver tissue (Supplementary Figure s1). To investigate the physiological function of EVA1A in the liver, we generated liver-specific *Eva1a* KO mice. The *Eva1a* flox/flox mice strain contains two loxp sequences which flank exon 3 of the mouse *Eva1a* gene and a neo cassette. An Alb-Cre-mediated deletion led to a deletion mutation due to the direct splicing from exon 3 and the neo cassette, producing a small truncated and nonfunctional peptide (Fig. 2a). A previous study showed that intact EVA1A is required for its biological activities and that the N-terminal of EVA1A fails to induce cellular autophagy and apoptosis^19^.

The resulting *Eva1a* flox/flox: Alb-Cre (*Eva1a*^-/-^) mice do not produce a spontaneous phenotype from age-matched control *Eva1a* flox/flox *(Eva1a*^+/+^) littermates. *Eva1a*^+/+^ and *Eva1a*^-/-^ mice were identified by PCR analysis of mouse tail DNA (Fig. 2b). *Eva1a*^*−/−*^ *m*ice were identified by RT-PCR, Western blot, and immunohistochemistry analysis, and revealed an *Eva1a* deletion in the *Eva1a*^*−/−*^ liver (Fig. 2c–e). We also evaluated the level of *Eva1a* mRNA expression in other tissues (e.g., brain and heart). There was no significant change between the *Eva1a*^+/+^ and *Eva1a*^-/-^ mice (Fig. 2c) in these tissues, indicating that the *Eva1a* deficiency was restricted to the liver.

### Deletion of *Eva1a* aggravates hepatic damage in ALF mice

We first studied whether the *Eva1a*^−/−^ mice affected normal liver function. Unfortunately, there were no obvious differences between the *Eva1a*^*+/+*^ and *Eva1a*^*−/−*^ mice in the appearance of the liver, the levels of alanine aminotransferase (ALT), and aspartate aminotransferase (AST) in the mouse serum.

**Fig. 3:**
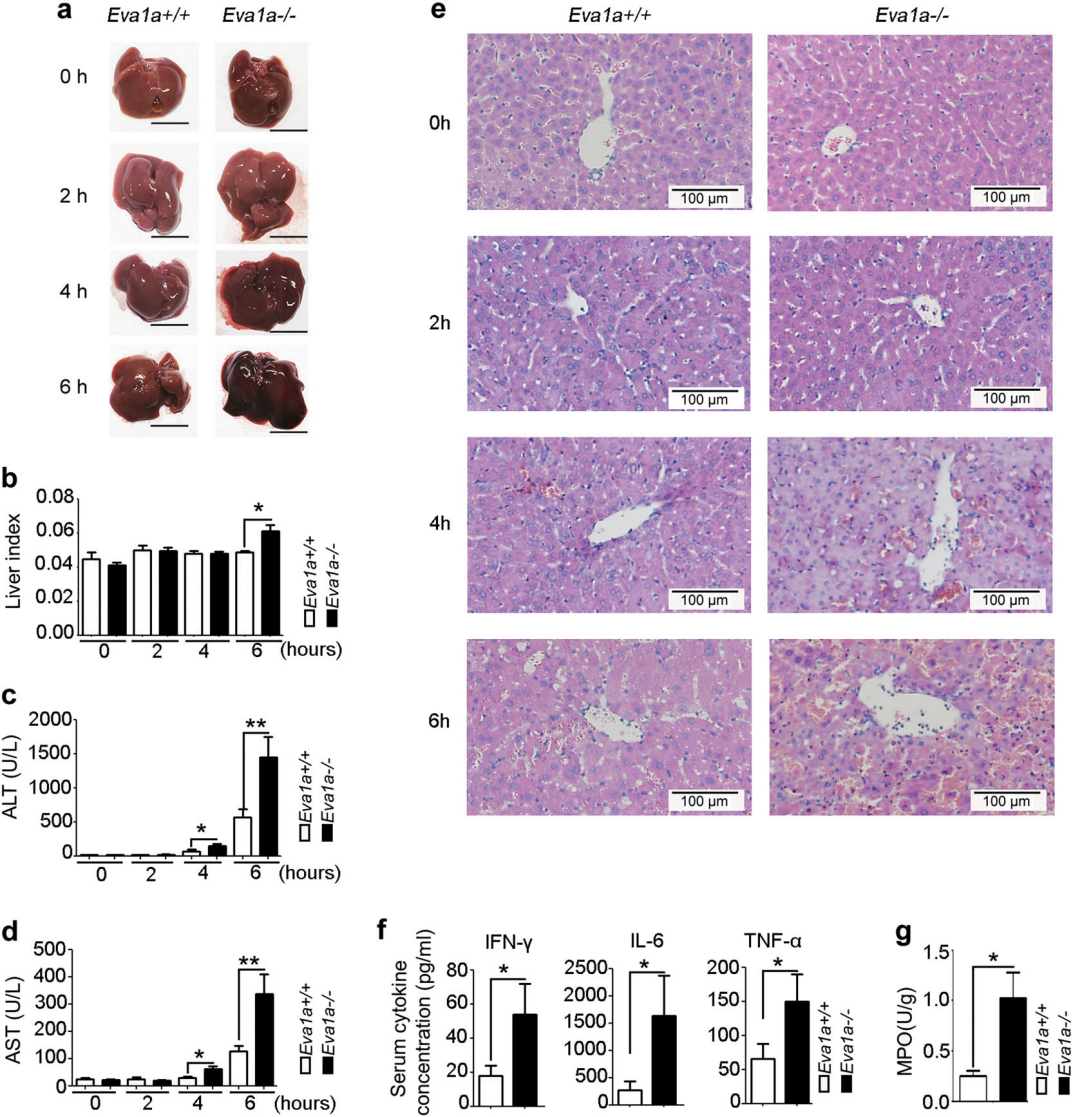
Genetic deletion of *Eva1a* aggravates hepatic injury in ALF mice. **a** Representative livers from different groups. Scale bar = 1 cm. **b** The liver index (the ratio of liver weight to body weight) of mice in different groups (*P < 0.05, *n* ≥ 4). **c**, **d** Serum ALT and AST levels from different groups (*P < 0.05, **P < 0.01, *n* ≥ 4). **e** Representative micrographs of livers stained with H&E from different groups. Scale bar = 100 µm. **f** The levels of IFN-γ, IL-6, and TNF-α in the serum from different groups at 6 h after treatment with d-GalN/LPS (*P < 0.05, *n* ≥ 10). **g** MPO levels in livers 6 h after treatment with d-GalN/LPS (*P < 0.05, *n* = 5)

We next investigated whether the *Eva1a* deletion was associated with d-GalN/LPS-induced ALF. From the observations of the gross morphology of the liver, we found that the *Eva1a*^−/−^ livers displayed spotty hemorrhaging at 4 h and total liver congestion at 6 h, whereas the *Eva1a*^+/+^ liver exhibited only partial hemorrhaging at 6 h (Fig. 3a). The liver index (the liver weight/body weight) of the *Eva1a*^−/−^mice was significantly increased at 6 h compared that of the *Eva1a*^+/+^ mice (Fig. 3b). The levels of serum ALT and AST in the *Eva1a*^−/−^ mice increased significantly at 4 h and 6 h compared with the *Eva1a*^+/+^ mice (Fig. 3c, d). The hematoxylin and eosin (H&E) staining results were in agreement with the increased serum ALT and AST levels (Fig. 3e). These data suggest that the deletion of *Eva1a* aggravates the severity of liver injury in ALF mice.

To determine the impact of the inactivation of *Eva1a* on the induction of inflammatory cytokines by d-GalN/LPS-induced ALF, the serum and liver tissues were harvested at 6 h following a d-GalN/LPS injection. As shown in Fig. 3f, the levels of inflammatory cytokines (e.g., TNF-α, IL-6, and IFN-γ) significantly increased in the serum of *Eva1a*^−/−^ mice compared with that of *Eva1a*^+/+^ mice (Fig. 3f). Consistent with the findings in the serum, inflammatory cytokines were also increased in the liver tissues of *Eva1a*^−/−^ mice compared with that of *Eva1a*^+/+^ mice (Supplementary Figure 2a). RT-PCR data showed that the levels of *IL6* and macrophage chemotactic protein-1 (*MCP1*) mRNA expression were upregulated *in Eva1a*^−/−^ livers compared with that of *Eva1a*^+/+^ livers (Supplementary Figure 2b).

We also detected myeloperoxidase (MPO) activity in liver tissues as a measure of neutrophil infiltration. As shown in Fig. 3g, the deletion of *Eva1a* significantly increased MPO activity-related injury in ALF induced by d-GalN/LPS. Taken together, these results indicate that the knockout of *Eva1a* enhanced d-GalN/LPS-induced ALF, which was associated with increased hepatic inflammation.

### Deletion of *Eva1a* results in mitochondrial damage and increased apoptosis in mice with ALF

**Fig. 4:**
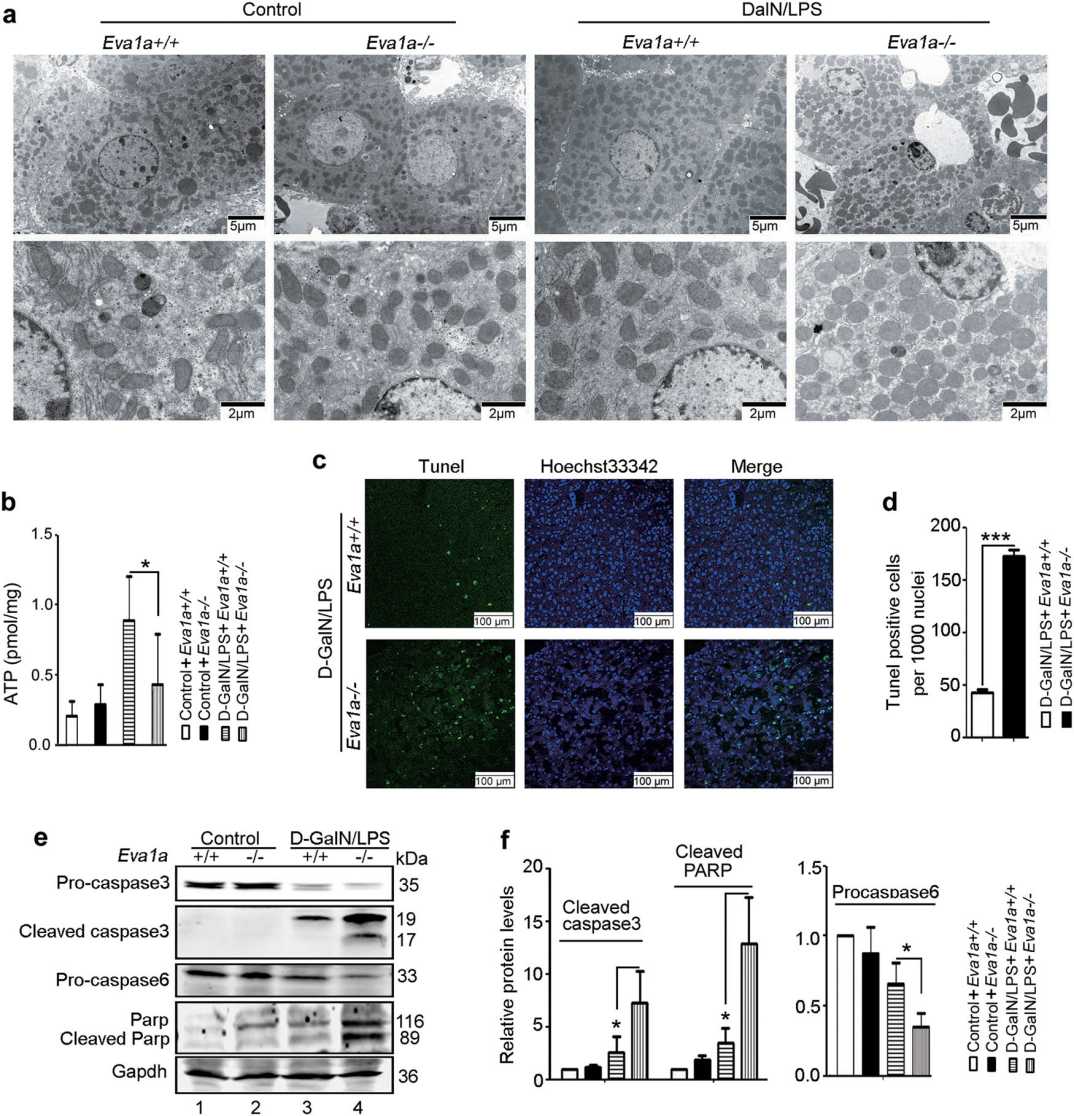
Deletion of *Eva1a* results in mitochondrial damage and increased apoptosis in mice with ALF. **a** Ultrastructural images reveal the accumulation of swollen mitochondria in d-GalN/LPS treated *Eva1a*^−/−^ mice. Scale bar = 5 µm or 2 µm. **b** Detection of ATP levels in the livers from different groups (*P < 0.05, *n* = 3). **c** Representative images of TUNEL staining (green) and Hoechst staining (blue) of the nuclei in liver tissues. Scale bar = 100 µm. **d** Quantification of TUNEL-positive cells per 1000 nuclei (***P < 0.001). **e** Representative Western blot of Pro-caspase 3, Cleaved caspase 3, Pro-caspase 6 and Parp in the liver extracts obtained from different groups of mice. **f** Quantification of indicated protein levels relative to Gapdh treated as described in **e**. Average value of *Eva1a*^+/+^ mice without d-GalN/LPS was normalized to 1 (*P < 0.05, *n* ≥ 3)

Using transmission electron microscopy (TEM), we analyzed the liver structure in normal mice and those with ALF. Under normal conditions, there was no significant difference in the mitochondria between *Eva1a*^+/+^ and *Eva1a*^−/−^ mice. Following treatment with d-GalN/LPS, *Eva1a*^−/−^ mice displayed many swollen mitochondria in liver compared with *Eva1a*^+/+^ mice (Fig. 4a). Our results were similar to those of previous studies, in which that the loss of *Eva1a* led to disorganized mitochondria in the heart^22^. Since the liver is rich in mitochondria, which is involved in energy metabolism, we wondered whether the deletion of *Eva1a* led to the lack of ATP generation. The experimental results revealed that *Eva1a*^*−/−*^ ALF mice exhibited a significantly lower ATP levels than that of *Eva1a*^+/+^ mice (Fig. 4b). These results indicated that the *Eva1a* deletion led to mitochondrial damage, accompanied by an inhibition of the clearance of damaged mitochondria.

It has been established that the loss of mitochondrial function results in cell death and various types of diseases^24^. We next investigated hepatocyte apoptosis in d-GalN/LPS-induced ALF. We performed a terminal deoxynucleotidyl transferase-mediated dUDP nick-end labeling (TUNEL) assay and found that there was a significantly higher proportion of apoptotic cells in the *Eva1a*^−/−^ mice with ALF than that in the *Eva1a*^+/+^ mice (Fig. 4c, d). We also examined caspase 3 and caspase 6, which act as lethal proteases at the most distal stage of the apoptotic pathway^25^. Our results revealed that the levels of cleaved caspase 3 was significantly higher in d-GalN/LPS-treated *Eva1a*^−/−^ mice than that in *Eva1a*^+/+^ mice (Fig. 4e, f and Supplementary Figure 3). The level of pro-caspase 3 and pro-caspase 6 were lower in *Eva1a*^−/−^ mice with ALF than that in *Eva1a*^+/+^ mice (Fig. 4e, f). Poly ADP-ribose polymerase (Parp) is one of the substrates of activated caspase 3^25^. It was found that there was a significant accumulation of cleaved Parp in *Eva1a*^−/−^ mice with ALF than that in *Eva1a*^+/+^ mice (Fig. 4e, f). These data suggest that an *Eva1a* deficiency is capable of increasing hepatocyte apoptosis in mice with hepatic failure induced by d-GalN/LPS.

### Genetic deletion of *Eva1a* impairs autophagy in mice with ALF

**Fig. 5:**
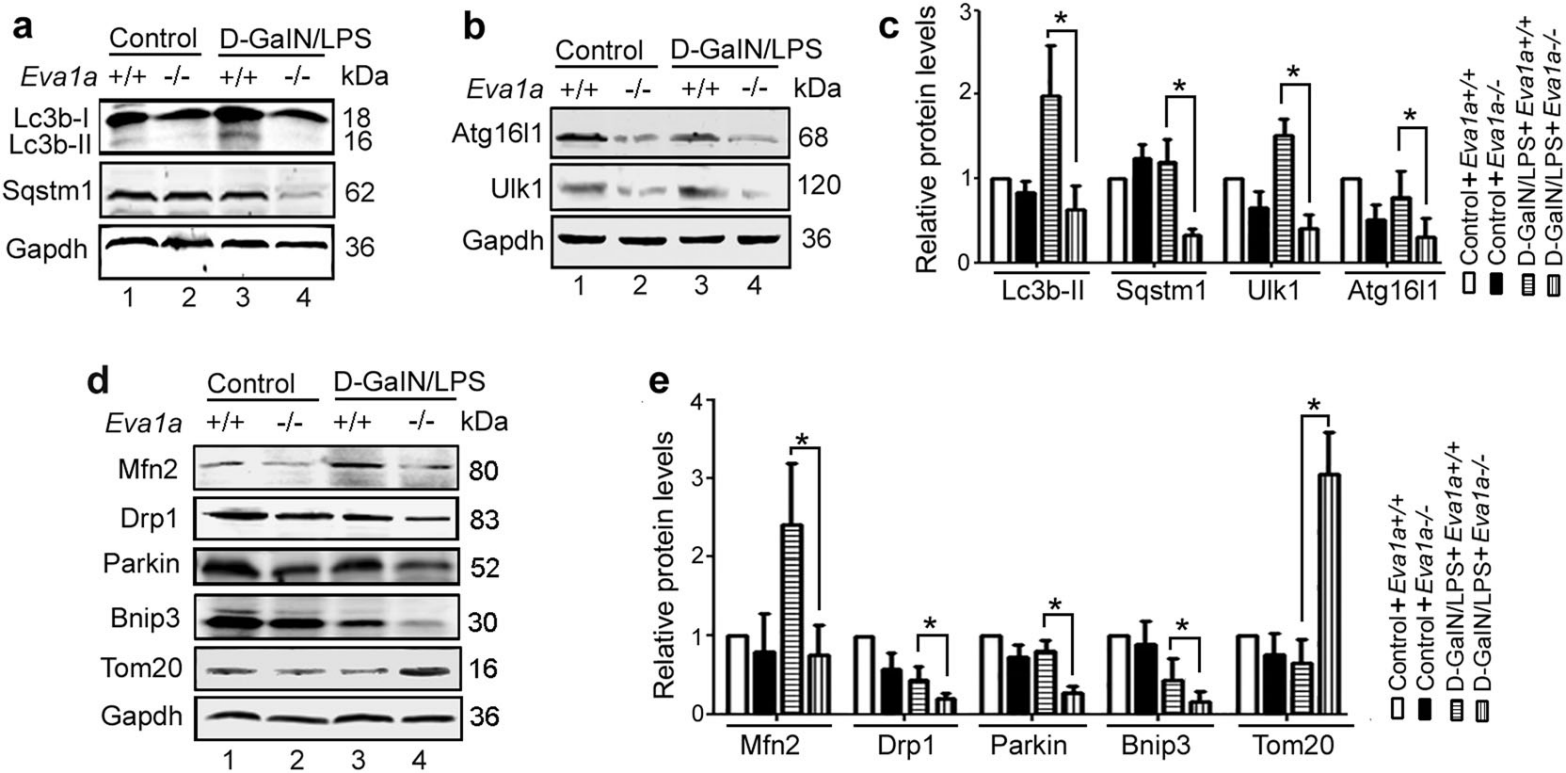
Deletion of *Eva1a* impairs autophagy in mice with ALF. **a**, **b** Representative Western blot of autophagy-related proteins Lc3b, Sqstm1, Ulk1, and Atg16l1 in the liver extracts obtained from different groups of mice. **c** Quantification of indicated protein levels relative to Gapdh treated as described in **a,** **b**. Average value for *Eva1a*^+/+^ mice without d-GalN/LPS was normalized to 1(*P < 0.05, *n* = 3). **d** Representative Western blot of Mfn2, Drp1, Parkin, Bnip3, and Tomm20 in the liver extracts obtained from different groups of mice. **e** Quantification of indicated protein levels relative to Gapdh treated as described in **d**. Average value for *Eva1a*^+/+^ mice without d-GalN/LPS was normalized to 1 (*P < 0.05, *n* ≥ 3)

Previous studies have shown that Eva1a-mediated autophagy maintains cellular functions in different tissues^21,22^. Thus, we asked whether Eva1a-mediated autophagy protects mice from d-GalN/LPS-induced ALF. To this end, we examined the level of autophagy in the liver of *Eva1a*^−/−^ mice. Under normal conditions, there was no obvious change in the levels of autophagy between *Eva1a*^+/+^ and *Eva1a*^−/−^ mice. The Western blot analysis showed that the accumulation of Lc3b-II was lower in *Eva1a*^*−/−*^ livers from mice with ALF compared to those of *Eva1a*^+/+^ mice (Fig. 5a–c). It has been reported that the Sqstm1 and Nbr1 serve as a receptors that are involved in completed autophagosome and are degraded in lysosomes^10^. A Western blot and immunohistochemical analysis revealed that the levels of Sqstm1 protein were lower in the livers of *Eva1a*^−/−^ mice with ALF than that of *Eva1a*^+/+^ mice (Fig. 5a–c, and Supplementary Figure 4). Additionally, the data from immunohistochemical analysis indicated that the staining intensity of Nbr1 and ubiquitin were also weaker in *Eva1a*^−/−^ ALF than that in *Eva1a*^+/+^ mice (Supplementary Figure 4), indicating the damage of autophagy in *Eva1a*^−/−^ mice with ALF.

Since it was previously reported that EVA1A interacts with Atg16l1 and induces autophagosome formation^19^, we examined the level of Atg16l1 in the livers of *Eva1a*^−/−^ mice. It was found that the levels of Atg16l1 and Ulk1 were downregulated in *Eva1a*^−/−^ mice with ALF (Fig. 5b, c). These results demonstrate that autophagy induced by Eva1a may play an important protection role against d-GalN/LPS-induced ALF.

Since the deletion of *Eva1a* inhibited the clearance of damaged mitochondria (Fig. 4a), and mitophagy plays an important role in removing damaged mitochondria and maintaining metabolic function^26^, so we next analyzed the levels of some mitophagy-related proteins. As shown in Fig. 5d, e, the levels of Mitofusin2 (Mfn2, mediates mitochondrial fusion), Drp1 (mediates mitochondrial division), Bnip3 and Parkin (mediates mitophagy)^26^, were decreased in *Eva1a*^−/−^ mice with ALF (Fig. 5d, e). In addition, the mitochondrial marker, Tomm20, was increased in *Eva1a*^−/−^ mice with ALF (Fig. 5d, e). Integrating the results of Fig. 4a, Fig. 5, and Supplementary Figure 4, we concluded that the loss of *Eva1a* may lead to a reduction of molecules (such as Sqstm1 and ubiquitin) required for autophagosome formation, following the decrease in the clearance of damaged mitochondria, and finally cellular apoptosis or death. Thus, Eva1a-mediated autophagy may contribute to the suppression of liver inflammation and maintain mitochondrial homeostasis in the context of ALF.

### Rapamycin and AAV-Eva1a protect against liver injury in *Eva1a*^−/−^ mice with ALF

**Fig. 6:**
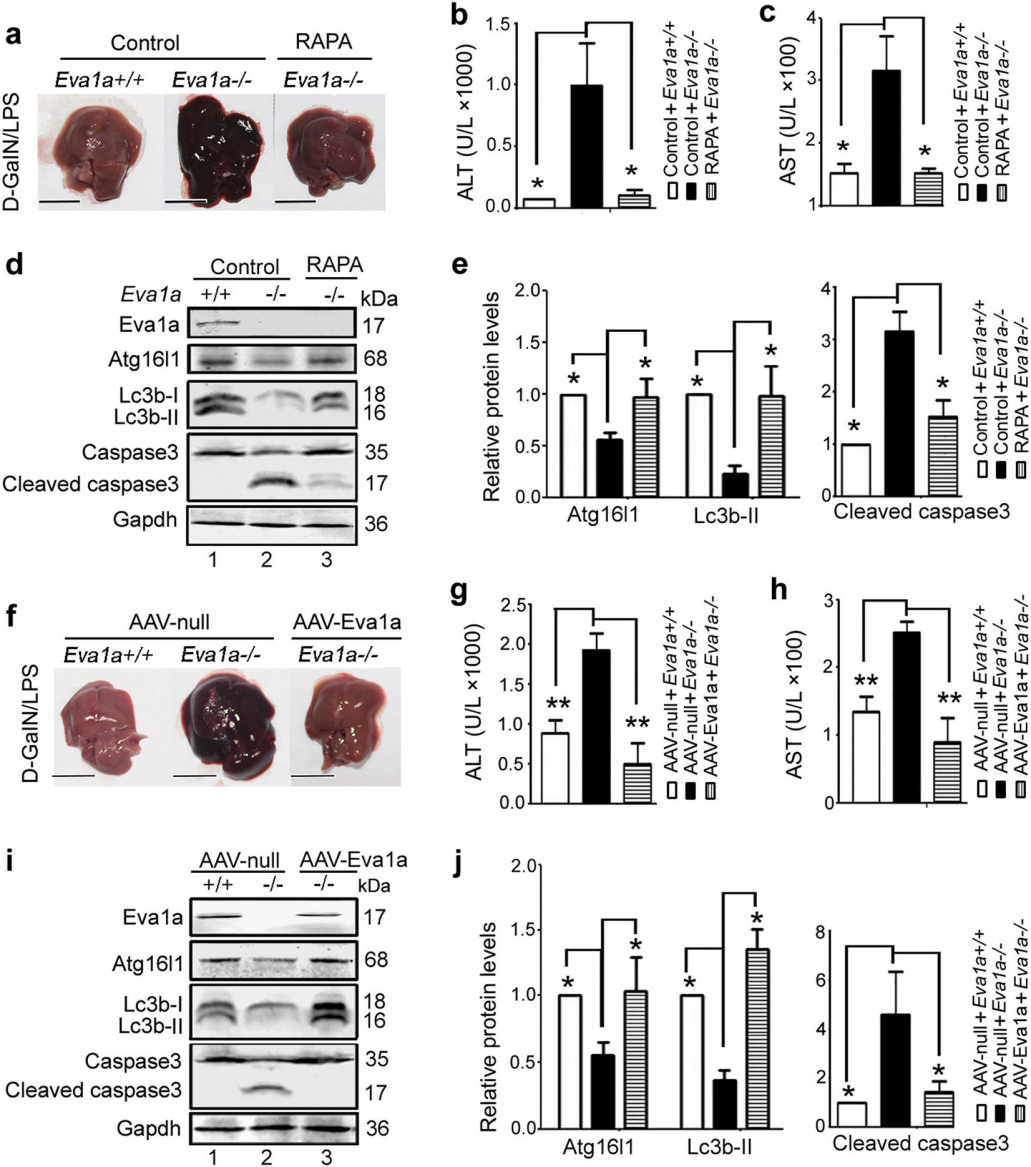
Rapamycin and AAV-Eva1a protect against liver injury in *Eva1a* KO mice with ALF. **a** Representative images of the livers from different groups. Scale bar = 1 cm. **b**, **c** Serum ALT and AST enzyme levels from different groups (**P < 0.01, *n* = 5). **d** Representative Western blot of Lc3b, Cleaved Caspase 3, Atg16l1, and Eva1a in the liver extracts obtained from different groups of mice. **e** Quantification of indicated protein levels relative to Gapdh treated as described in **d**. Average value for *Eva1a*^+/+^ mice was normalized to 1 (*P < 0.05, *n* = 3). **f** Representative image of the livers from different groups. Scale bar = 1 cm. **g**, **h** Serum ALT and AST enzyme levels from different groups (**P < 0.01, *n* = 3). **i** Representative Western blot of Atg16l1, Lc3b, Cleaved Caspase 3, and Eva1a in the liver extracts obtained from different groups of mice. **j** Quantification of indicated protein levels relative to Gapdh treated as described in **i**. Average value for *Eva1a*^+/+^ mice was normalized to 1 (*P < 0.05, *n* = 3)

Since the downregulation of autophagy led to severe liver injury in mice with ALF, we wondered if increased autophagy could alleviate the liver damage associated with ALF. We first injected rapamycin (RAPA, inhibitor of MTOR) into *Eva1a*^−/−^ to induce autophagy. The *Eva1a*^−/−^ mice presented with severe liver injury with ALF compared with that *Eva1a*^+/+^ mice (Fig. 6a−c). In contrast, in *Eva1a*^−/−^ mice pretreated with rapamycin, d-GalN/LPS-induce liver injury was significantly suppressed, evidenced by decreased hemorrhaging (Fig. 6a), and descended levels of serum ALT and AST (Fig. 6b, c) compared with that *Eva1a*^−/−^ mice only. At the same time, there was an accumulation of Lc3b-II and Atg16l1, whereas caspase 3 cleavage was blocked in rapamycin-pretreated *Eva1a*^−/−^ mice with ALF compared with that *Eva1a*^−/−^ mice only (Fig. 6d, e). Further investigates indicated that rapamycin could decrease the Tomm20 levels in *Eva1a*^−/−^ ALF (Supplementary Figure 5), implying that rapamycin-induced autophagy may enhance the clearance of damaged mitochondria.

Based on the above observations, we next performed reciprocal Eva1a gain-of-function experiments using an AAV-Eva1a construct or AAV-Null control. Four weeks following the AAV-Eva1a injection, the d-GalN/LPS-induced ALF model was performed. As shown in Fig. 6f, compared with AAV-Null injected *Eva1a*^−/−^ mice, the gross morphology of the liver in the AAV-Eva1a group appeared normal and the liver architecture was preserved (Fig. 6f). Similar to the rapamycin-treated mice, the recovery of Eva1a in *Eva1a*^−/−^ mice could decrease serum ALT and AST levels (Fig. 6g, h), increase the levels of Lc3b-II and Atg16l1, and attenuate the cleavage of caspase 3 in ALF mice (Fig. 6I, j). These results indicated that Eva1a protects mice against d-GalN/LPS-induced ALF by promoting autophagy.

## Discussion

EVA1A, also termed TMEM166 or FAM176A, was first characterized as an autophagy-related protein in our lab^14,16^. Previous studies have demonstrated that EVA1A can interact with ATG16L1 and promote autophagosome formation and programmed cell death^19^. In the present study, we investigated the role of *Eva1a* in mice with ALF induced by d-GalN/LPS. We demonstrated that Eva1a protects mice from ALF by modulating autophagy. The genetic deletion of *Eva1a* disrupted autophagosome formation, leading to the accumulation of damaged mitochondria, a decrease in ATP content, consequently promoting hepatocytes apoptosis, inflammation, and the aggravation of liver injury. This effect could be rescued by the overexpression of Eva1a or pretreatment with rapamycin. Our results suggest that Eva1a-mediated autophagy may be a protective factor in the context of mouse acute liver injury.

Several studies have suggested that autophagy is involved in acute liver injury^1,13,27–29^; however, whether autophagy is hepatoprotective remains controversial. Some studies have shown that autophagy limits inflammasome-associated pro-inflammatory cytokine maturation in ALF and the inhibition of hepatocyte autophagy increases liver injury by promoting caspase 8 activation and decreasing Akt levels^11,12,30^. NAADP-mediated Ca^2+^ signaling or peroxisome proliferator-activated receptor α activation promotes autophagy and protects mice from ALF. Moreover, the inhibition of glycogen synthase kinase 3β can also promote autophagy and decrease liver injury associated with ALF^1,28^. Enhancing autophagy by rapamycin ameliorates hepatotoxicity and the inhibition of autophagy following treatment with 3-MA or *Atg7* siRNA increases ALF liver injury^1^. In contrast, another report suggested that pretreatment with wortmannin (a PI3KC3 inhibitor) alleviates liver injury associated with ALF by attenuating autophagy^13^. Our study proved that Eva1a-mediated autophagy protected mice from ALF, supporting the notion that autophagy pathway is essential for protection against ALF.

Studies have found that mitochondrial dysfunction is involved in d-GalN/LPS-induced ALF. Functional mitophagy is essential for preventing the accumulation of abnormal or damaged mitochondria^26^. This process involves a number of molecules, including DRP1, MFN2, PRKN/Parkin, and BINP3. DRP1 is located in the mitochondrial outer membrane, generates small mitochondria, and allows the phagophore to efficiently engulf the organelle^31,32^. The mitochondrial protein, MFN2, mediates Parkin recruitment to damaged mitochondria^33^. Ubiquitination of outer mitochondrial membrane proteins by Parkin, an E3 ubiquitin ligase, induces the localization of target proteins to the phagophore through their interaction with p62/SQSTM1^10^. It has been reported that Bnip3, a BH3-only protein primarily localized to the mitochondria, interacts with LC3 to selectively remove dysfunctional mitochondria via autophagy^34^. In our observations, we found that the levels of Drp1, Mfn2, Parkin, and Bnip3 were decreased in the livers of *Eva1a*^−/−^ mice with ALF, indicating an impairment of mitophagy. Therefore, the damaged mitochondria failed to be degraded and consequently resulted in the accumulation of swollen mitochondria and increased hepatocyte apoptosis in *Eva1a*^−/−^ mice. This finding suggests that Eva1a may be involved in mitophagy to maintain mitochondrial homeostasis, for which the mechanism requires further investigation in the future.

Ubiquitin plays an important role for the autophagic removal of protein aggregates and damaged organelles. This is primarily achieved through several adapter molecules, (e.g., p62/SQSTM1 and NBR1), which can directly interact with poly- and mono-ubiquitin and LC3^10^. Our research found that in the liver of *Eva1a*^−/−^ mice with ALF, the levels of the autophagy markers, Lc3b-II, ubiquitin, p62/Sqstm1, and Nbr1, were significantly decreased compared to that of the *Eva1a*^+/+^ liver. Other researchers also observed that impaired autophagy in d-GalN/LPS-induced ALF mice was companied with the reduction of p62/Sqstm1 levwls^27^. Additionally, *Eva1a* deletion downregulates the levels of Atg16l1 and Atg12-Atg5, which are required for the extension of the isolation membrane. Since the reduction of these autophagic elements might impact the formation of the autophagosome^1,27^, such damaged mitochondria or other aggregates were not enveloped by autophagosomes in the absence of EVA1A and failed to be degraded in lysosomes. In this manner, cellular apoptosis or death is inevitable.

Rapamycin is an inhibitor of the mammalian target of rapamycin (MTOR) signaling pathway, which regulates cell growth, protein synthesis, and autophagy. It is reported that pretreatment of rapamycin protects against d-GalN/LPS-induced ALF via autophagy^1^. Our present results were consistent with this phenotype, which supporting the functional connection among rapamycin, autophagy and ALF. Based on the fact that rapamycin has multiple effects, such as immunosuppressive^35^ and anticancer activities^36,37^, we further performed reciprocal Eva1a gain-of-function experiments to explore the functional correlation between autophagy and ALF. Our data proved that the recovery of Eva1a in *Eva1a*^*−/−*^ mice could increase autophagy and decrease the damage of hepatocytes, suggesting that Eva1a-mediated autophagy protects mice against acute liver injury.

In summary, our findings provide insight into the activities of Eva1a, as well as the role of autophagy in acute liver injury. Our study identifies that the promotion of autophagy may be a potential therapeutic target for ALF, which is consistent with other studies. Further preclinical studies on autophagy-inducing therapies are expected.

## Materials and Methods

### Antibodies and reagents

The following antibodies were used: Rabbit anti-Eva1a/Tmem166 (GeneTex, Irvine, CA, USA, GTX32925), Rabbit anti-Lc3b (Sigma Aldrich, St. Louis, MO, USA, L7543), Mouse anti-Gapdh (Sungene, Tianjin, China, KM9002). Antibodies against Atg5 (12994), caspase3 (39665), cleaved caspase3 (39664), Parp (9532), and Ubiquitin (3936) were purchased from Cell Signaling Technology (Danvers, MA, USA). Antibodies against Ulk1 (ab128859), Atg16l1 (ab187671), Nbr1 (ab55474), Caspase 6 (ab185645), Bnip3 (ab109362), Drp1 (ab184247), Mfn2 (ab124773), Parkin (ab179812), and Tomm20 (ab186734) were purchased from Abcam (Cambridge, UK). Antibodies against p62/SQSTM1 (PM045) and Beclin1 (PD017) were purchased from MBL International (Woburn, MA, USA). Secondary antibodies included DyLight 800/DyLight 680-conjugated IgG against mouse (Rockland, Philadelphia, PA, USA, 610-145-002/610-144-002) or rabbit (Rockland, 611-145-002/611-144-002).

### Generation of liver-specific Eva1a-deficient mice

*Eva1a*^*flox/flox*^ mice on a C57BL/6 background were constructed by the Model Animal Research Center of Nanjing University (Nanjing, China). Alb-Cre transgenic mice were provided by Shanghai Biomodel Organism Science and Technology Development Co., Ltd., Shanghai, China. Progeny containing the *Eva1a* flox allele were mated with Cre transgenic mice to generate *Eva1a*^flox/flox; Alb-Cre^ mice. All mice used in the study were bred and maintained at the Experimental Animal Center, Peking University Health Sciences Center (Beijing, China) under a 12-h light/dark cycle. All mice were given free access to water and standard mouse chow. The animal experiment protocol was approved by the Biomedical Research Ethics Committee of Peking University and strictly adhered to the American Physiological Society’s Guiding Principles in the Care and Use of Vertebrate Animals in Research and Training.

### Animal model

Male C57BL/6 mice (8–12 weeks old) were intraperitoneally (i.p.) injected with D -GalN (350 mg/kg; Sigma, St. Louis, MO, USA) and LPS (30 μg/kg; Sigma, St. Louis, MO, USA) to induce ALF. Control mice received the same volume of PBS. To induce autophagy, rapamycin (2 mg/kg; Sigma) were given i.p. to mice 2 h before the administration of d-GalN/LPS.

For the rescue assay, AAV-Eva1a (Likely Biotechnology, Beijing, China) was injected via the tail vein to mice four weeks before the injection of d-GalN/LPS, and AAV-null was used as a control. The mice were killed at various time points following d-GalN/LPS treatment, and the liver tissue and serum samples were collected for future analysis.

### Genotype analysis by PCR

*Eva1a* genotyping was performed by PCR using DNA isolated from the tails of mice. Mouse tails were soaked in 100 μL of 25 mM NaOH and 0.2 mM EDTA lysis buffer, heated for 30 min at 95 °C, and neutralized using 100 μL of 40 mM Tris-HCl (pH 5.5). Supplemental Table 1 presents a list of the primers used for genomic PCR.

### Serum ALT, AST, and liver MPO analysis

Serum ALT and AST were measured using commercial diagnostic kits (Nanjing Jiancheng Bioengineering Institute, Nanjing, China, C009-3/C010-3) following the manufacturer’s instructions. Liver MPO levels were detected using diagnostic kits (Nanjing Jiancheng Bioengineering Institute, Nanjing, China, A044) in accordance with the manufacturer’s instructions.

### RNA isolation and real-time RT-PCR

Total RNA was prepared from mouse tissues using TRIZOL reagent (Invitrogen, Carlsbad, CA, USA; 15596-026), and cDNA was synthesized using a Revert Aid First Strand cDNA Synthesis Kit (Thermo Scientific, Waltham, MA, USA; C28025-032). mRNA levels were analyzed by RT-PCR or quantitative RT-PCR (qRT-PCR) and normalized to the levels of the Gapdh housekeeping gene. RT-PCR and qRT-PCR assays were performed in triplicate for each sample. Supplemental Table 1 presents a list of the primers used for RT-PCR and qRT-PCR.

### Western blot analysis

The total protein from mouse tissues were extracted using RIPA lysis buffer (50 mM Tris [pH 7.4], 150 mM NaCl, 1% NP-40, 0.5% sodium deoxycholate, 0.1% SDS, Beyotime, Shanghai, China) containing a freshly added proteinase inhibitor cocktail (Roche Diagnostics, Berlin, Germany). Protein concentrations were determined using a BCA protein assay reagent (Beyotime, Shanghai, China; P0010). Equal amounts of proteins were separated by SDS-PAGE electrophoresis and transferred to nitrocellulose membranes. After blocking with 5% nonfat milk for 1 h, the membranes were incubated with the primary antibodies overnight at 4 °C, washed, and then incubated with the DyLight 800/DyLight 680-conjugated secondary antibodies. The membranes were then washed and scanned using an Odyssey Infrared Imaging System (LI-COR Biosciences, Lincoln, NE, USA). The scanned bands were quantified using ImageJ software. The results were representative of at least three experiments.

### Histological and immunohistochemical analysis

The liver tissues were fixed overnight in 4% paraformaldehyde, dehydrated in a graded series of ethanol, and embedded in paraffin. In the histopathological analysis, 2.5 μm sections were stained with H&E using standard procedures.

For the immunohistochemical analysis, sections were deparaffinized and rehydrated. Antigen retrieval was performed in a pressure cooker at 100 °C for 2 min in 0.01 M sodium citrate (pH 6.0), and endogenous peroxidase activity was blocked with 3% hydrogen peroxide. The slides were then incubated in 5% goat serum. Following an incubation with primary antibodies at 4 °C overnight and washing three times in PBS, the sections were conducted with a DAB Detection Kit (PV-6000-D, Origene, China) according to the manufacturer’s instructions. The sections were developed with a DAB substrate and counter-stained with hematoxylin. The samples were then dehydrated and sealed with coverslips.

TUNEL assays were performed using an in situ cell death detection kit (Roche Applied Science, Indianapolis, IN, USA) according to the manufacturer’s instructions. The sections were counterstained with Hoechst 33342 (Sigma Aldrich, 14533).

### Detection of cytokines in the serum

The concentration of cytokines in the serum were measured using the Multi-Analyte Flow Assay Kit for Mouse Th1/Th2 Panel (Biolegend, San Diego, CA, USA, 740029) according to the manufacturer’s instructions. The cytokine concentration in individual mouse liver tissue was also detected using the same method.

### Transmission electron microscopy

The liver tissues were initially fixed in 0.1 M sodium phosphate buffer containing 3% glutaraldehyde (pH 7.4) and then fixed in 0.1 M sodium phosphate buffer containing 1% OsO4 (pH 7.2) for 2 h at 4 °C. The tissues were dehydrated in a graded ethanol series, embedded in Ultracut (LEICA ULTRACUTR, Bensheim, Germany), and sliced into 60 nm sections. The ultrathin sections were stained with uranyl acetate and lead citrate and observed under a JEM-1230 transmission 855 electron microscope (JEOL-USA, Inc., Peabody, MA, USA).

### Detection of ATP levels

The ATP levels of liver tissues from mice were measured using a firefly luciferase-based ATP assay kit (Beyotime, Shanghai, China), according to the manufacturer’s instructions. After the indicated treatments, the liver tissues were homogenated and centrifuged at 12,000×*g* for 5 min. The supernatants (100 μL) were mixed with 100 μL of ATP detection solution at a working dilution in a white 96-well plate. Standard curves were also generated, and the protein concentration of each treatment group was determined using a Bradford protein assay. The total ATP levels were expressed as nmol/mg protein. This experiment was repeated three times.

### Statistical analysis

The data are presented as the means ± S.D. Differences between groups were compared using Prism 5 (GraphPad Software Incorporate, La Jolla, CA, USA) with Student’s *t-*test. A *P* value of <0.05 was considered statistically significant.

## Electronic supplementary material

Supplemental Table

Supplementary figure legends

Supplementary Figure 1

Supplementary Figure 2

Supplementary Figure 3

Supplementary Figure 4

Supplementary Figure 5
