# Supplementary material for: Liver-specific deletion of Eva1a/Tmem166 aggravates acute liver injury by impairing autophagy
Source: Cell Death Dis. 2018 Jul 10;9(7):768. doi: 10.1038/s41419-018-0800-x (PMC6039435; doi:10.1038/s41419-018-0800-x)
Supplement: Supplementary file 1 — Supplemental Table [file 41419_2018_800_MOESM1_ESM.docx]

| Supplemental Table 1. Primers used for genome PCR, RT-PCR, qRT-PCR |
| --- |
| \|  \| Forward Primer \| Reverse Primer \| \| --- \| --- \| --- \| \| Genome PCR \|  \|  \| \| *Eva1a-GT(5)* \| ATCTGTTAGGGACAAGGGTA \| CAAAGGAGAATGGCAAATGG \| \| *Eva1a-GT(3)* \| TCTGAGGCGGAAAGAACCAG \| CAGCCCAGGAAATAGGATGA \| \| *Cre* \| GCCTGCATTACCGGTCGATGC \| CAGGGTGTTATAAGCAATCCC \| \| RT-PCR \|  \|  \| \| *Eva1a* \| GCCGCTCTGTACTTTGTC \| TCTCCCTGATCATTCGTT \| \| *Gapdh* \| GACCACAGTCCATGCCATCAC \| TCCACCACCCTGTTGCTGTAG \| \| qRT-PCR \|  \|  \| \| *Eva1a* \| TTGGGAATGGCTCTGCTC \| CTCGCTCAGGGTTTTCTGA \| \| *Mcp1* \| TTTCGTCTCTAGCCGCGTG \| ACCACGCTGAAGGTGTTCAT \| \| *Il6* \| GCTACCAAACTGGATATAAT  CAGGA \| CCAGGTAGCTATGGTACTCCA  GAA \| \| *Il10* \| CATGGCCCAGAAATCAAGGA \| TGCTCCACTGCCTTGCTCTT \| \| *β-actin* \| CTAAGGCCAACCGTGAAAAG \| ACCAGAGGCATACAGGGACA \| |
