# Supplementary material for: Liver-specific deletion of Eva1a/Tmem166 aggravates acute liver injury by impairing autophagy
Source: Cell Death Dis. 2018 Jul 10;9(7):768. doi: 10.1038/s41419-018-0800-x (PMC6039435; doi:10.1038/s41419-018-0800-x)
Supplement: Supplementary file 2 — Supplementary figure legends [file 41419_2018_800_MOESM2_ESM.docx]

**Supplementary figure legends**

**Supplementary figure 1. *Eva1a* mRNA was expressed in different tissues of mice.** Representative RT-PCR results showing endogenous *Eva1a* mRNA in different tissues from wild-type mice.

**Supplementary figure 2. Deletion of *Eva1a* increases the levels of inflammatory cytokines in ALF mice**. **(a)** The levels of IFN-γ, IL-5, TNF-α, IL-6, IL-4, IL-10 and IL-13 in the D-GalN/LPS-treated liver tissues at 6 h from different groups (**P* < 0.05, n=5). **(b)** Quantitative RT-PCR analysis of the levels of *Il6*, *Il10* and *Mcp1* in the D-GalN/LPS-treated liver tissues at 6 h from different groups (**P* < 0.05, n=5).

**Supplementary figure 3.** **Deletion of *Eva1a* results in the activation of Caspase3 in ALF mice.** Representative images of immunohistochemical staining of cleaved Caspase3 in the D-GalN/LPS-treated liver tissues at 6 h from different groups. Isotype IgG staining was used as a negative control. Scale bar = 100 µm.

**Supplementary figure 4. Deletion of *Eva1a* results in a reduction of Sqstm1, Ubiquitin and Nbr1 in ALF mice.** Representative images of immunohistochemical staining of Sqstm1, Ubiquitin and Nbr1 in the D-GalN/LPS-treated liver tissues at 6 h from different groups. Isotype IgG staining was used as a negative control. Scale bar = 100 µm.

**Supplementary figure 5. Pretreatment of Rapamycin (RAPA) decreases the levels of Tomm20 in *Eva1a^-/-^* ALF.** (a) Representative Western blot of Tomm20 in the liver extracts obtained from different groups of mice. (b) Quantification of Tomm20 levels relative to Gapdh treated as described in (a). **P*<0.05.
