# Supplementary figures and images for: Liver-specific deletion of Eva1a/Tmem166 aggravates acute liver injury by impairing autophagy
Source: Cell Death Dis. 2018 Jul 10;9(7):768. doi: 10.1038/s41419-018-0800-x (PMC6039435; doi:10.1038/s41419-018-0800-x)

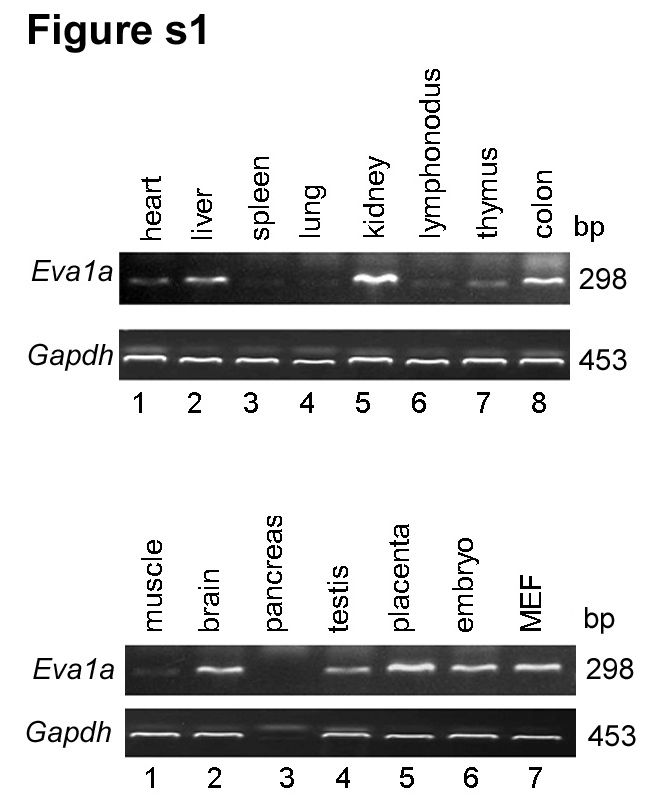

Supplement: Supplementary file 3 — Supplementary Figure 1 [file 41419_2018_800_MOESM3_ESM.jpg]

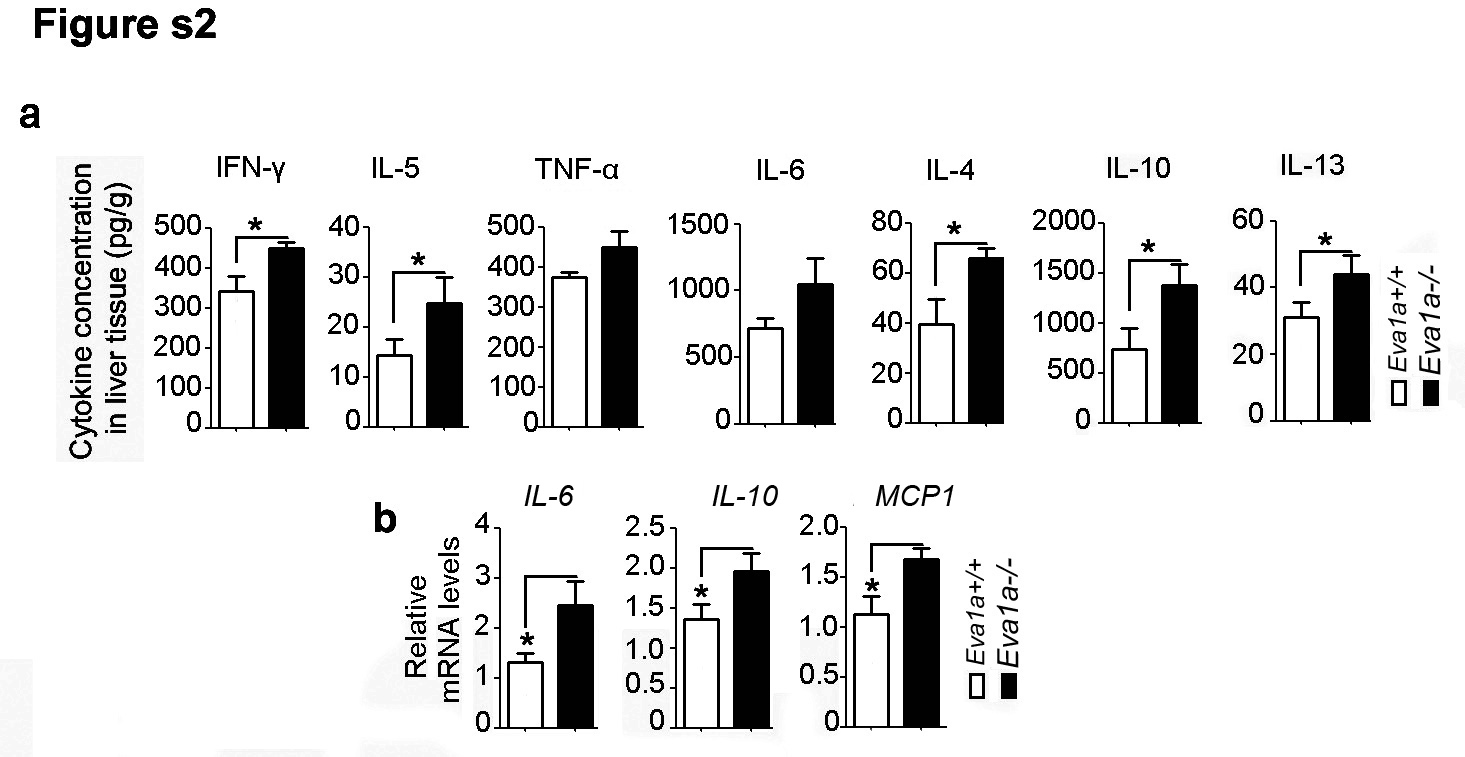

Supplement: Supplementary file 4 — Supplementary Figure 2 [file 41419_2018_800_MOESM4_ESM.jpg]

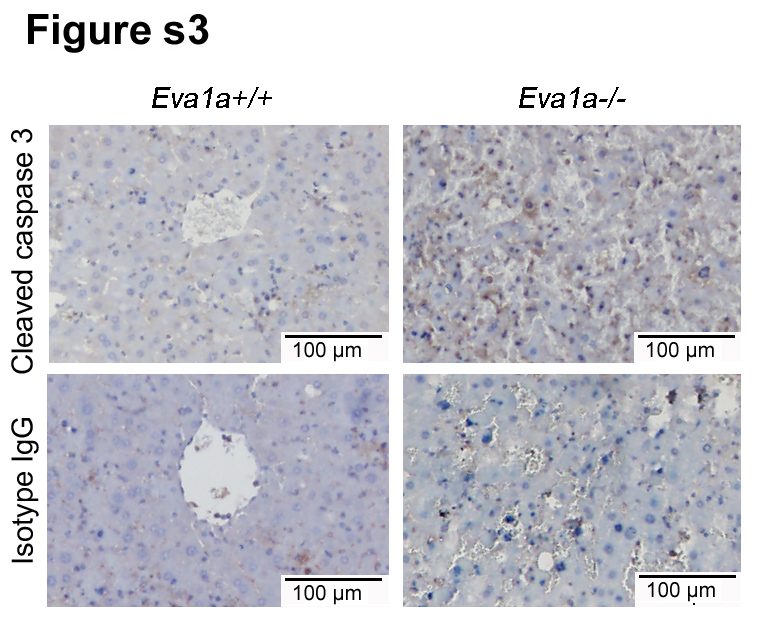

Supplement: Supplementary file 5 — Supplementary Figure 3 [file 41419_2018_800_MOESM5_ESM.jpg]

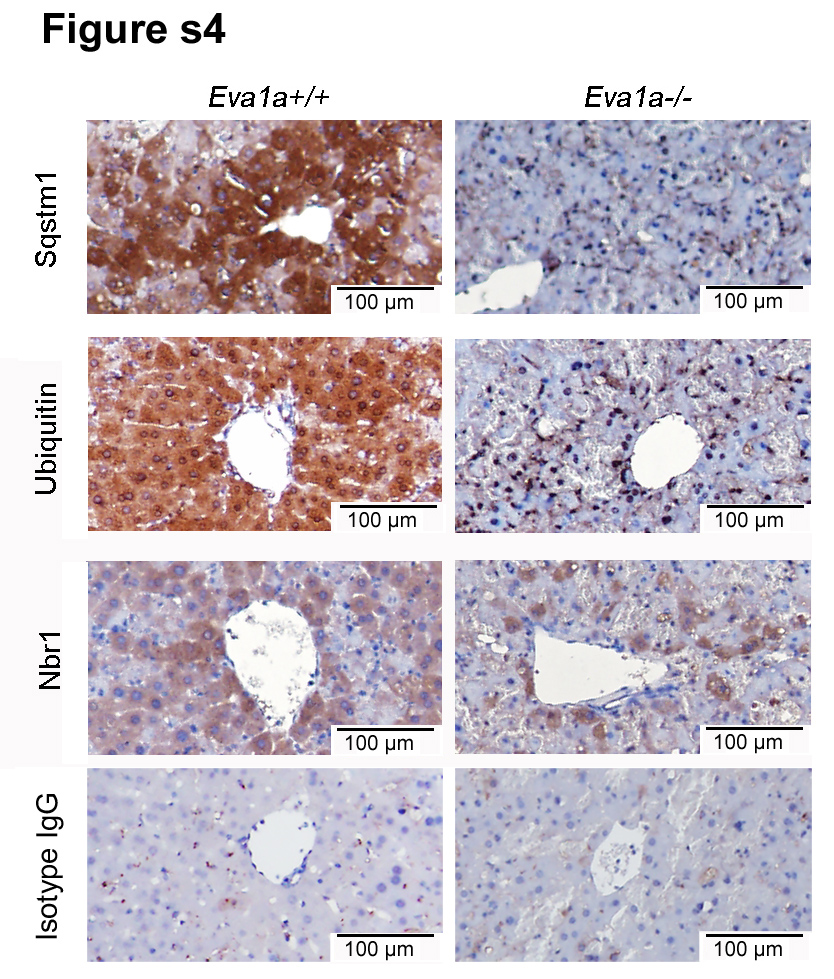

Supplement: Supplementary file 6 — Supplementary Figure 4 [file 41419_2018_800_MOESM6_ESM.jpg]

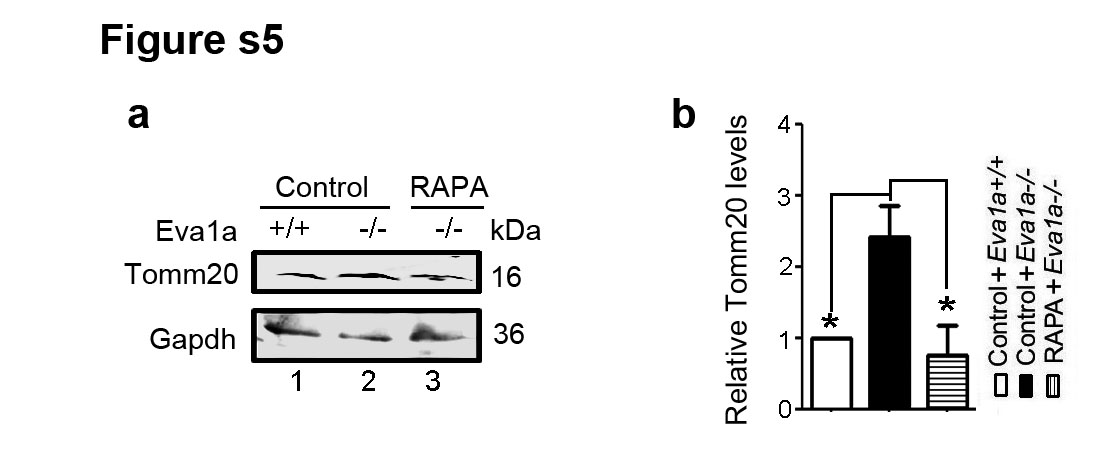

Supplement: Supplementary file 7 — Supplementary Figure 5 [file 41419_2018_800_MOESM7_ESM.jpg]
